# Electrocardiography is Useful to Predict Postoperative Ventricular Arrhythmia in Patients Undergoing Cardiac Surgery: A Retrospective Study

**DOI:** 10.3389/fphys.2022.873821

**Published:** 2022-05-02

**Authors:** Weichao Li, Weihua Liu, Heng Li

**Affiliations:** The Sixth Affiliated Hospital of Guangzhou Medical University, Department of Anesthesiology, Qingyuan People’s Hospital, QingYuan, China

**Keywords:** postoperative ventricular arrhythmia, electrocardiographic markers, model, J wave, abnormal repolarization

## Abstract

**Background:** Preoperative detection of high-/low-risk postoperative ventricular arrhythmia (POVA) patients using a noninvasive method is an important issue in the clinical setting. This study mainly aimed to determine the usefulness of several preoperative electrocardiographic (ECG) markers in the risk assessment of POVA with cardiac surgery.

**Method:** We enrolled 1024 consecutive patients undergoing cardiac surgery, and a total of 823 patients were included in the study. Logistic regression analysis determined preoperative ECG markers. A new risk predicting model were developed to predict occurrence of POVA, and the receiver operating characteristic curve (ROC) was used to validate this model.

**Results:** Of these, 337 patients experienced POVA, and 485 patients did not experience POVA in this retrospective study. Among 15 ECG markers, a univariate analysis found a strong association between POVA and preoperative VA, the R-wave in lead aVR, the QRS wave, index of cardiac electrophysiological balance (iCEB), QT interval corrected (QTc), Tpeak–Tend interval (Tpe) in lead V_2_, the J wave in the inferolateral leads, pathological Q wave, and S_V1_+R_V5_>35 mm. Multivariate analysis showed that a preoperative J wave [adjusted odds ratio (AOR): 3.80; 95% CI: 1.88–7.66; *p* < 0.001], Tpe >112.5-ms (AOR: 2.80; 95% CI: 1.57–4.99; *p* < 0.001), and S_V1_+R_V5_ >35 mm (AOR: 2.92; 95% CI: 1.29–6.60; *p* = 0.01) were independently associated with POVA. A new risk predicting model were developed in predicting POVA.

**Conclusion:** The ECG biomarkers including J wave, Tpe >112.5 ms, and S_V1_+R_V5_ >35 mm were significantly predicted POVAs. A risk predicting model developed with electrocardiographic risk markers preoperatively predicted POVAs.

## Introduction

Postoperative ventricular arrhythmia (POVA) is a frequent complication after surgery. Some clinical studies have suggested that POVA increases short- and long-term mortality and further deteriorates heart failure postprocedure (El-Chami et al., 2012; Valderrábano et al., 2016a). It is important to identify patients at high risk of developing POVA so that targeted prophylactic therapy or alternative strategies can be given. Many clinical markers for the development of malignant arrhythmia in ischemic heart diseases have been reported, including episodes of conduction disturbance postprocedure, T wave peak and end (Tpe) as an independent predictor of arrhythmic events (Maury et al., 2015), fragmented QRS (fQRS) (Morita et al., 2008), an early repolarization (ER) pattern (Pieroni et al., 2008), short- or long-QT intervals (Schwartz and Ackerman, 2013; Bjerregaard, 2018), the existence of late potential, and the index of cardiac electrophysiological balance (iCEB: QT/QRS) (Lu et al., 2013). In addition, the markers can also be used to stratify patients for short- and long-term outcomes and to predict sudden cardiac death due to malignant ventricular arrhythmia (SCD-VA) (Erikssen et al., 2012; Tse et al., 2018). However, the evidence on whether preoperative markers can predict an increased risk of POVA is not clear in the surgical setting. In this study, we chiefly aimed to identify preoperative risk markers for POVA by paying attention to surface electrocardiographic parameters.

## Methods

### Design, Setting, and Ethical Statement

Ethical approval for this retrospective cohort study (Ethical Committee approval number: IRB-202108-K4-amendment review-01) was provided by the Ethical Committee of the Sixth Affiliated Hospital of Guangzhou Medical University, QingYuan, GuangDong (Chairperson Prof JiFang Liu) on 12 November 2021. We registered the study protocol with the Chinese Clinical Trial Registration (identifier: ChiCTR2100052496).

### Data Source

The current study used the data archived in the electronic medical record system of Southern Huiqiao and in the anesthesia care system database in the operating room. The exact date of death of in-hospital patients was recorded explicitly.

### Study Population and Inclusion and Exclusion Criteria

**FIGURE 1:**
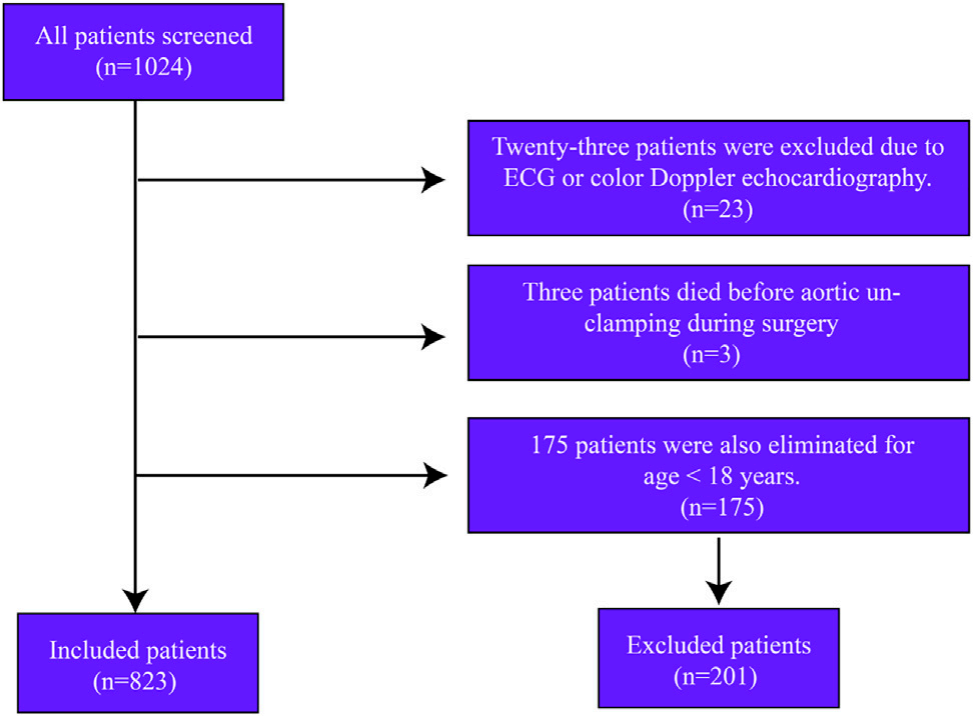
Flowchart of patient selection.

In this retrospective cohort study, we screened 1024 consecutive patients undergoing open cardiac surgery from January 2013 to October 2021. Twenty-three patients were excluded due to the loss of ECG data. Only three patients who died during surgery were excluded from the analysis. Another 175 patients were excluded for age <18 years. A total of 823 patients were included in the study (Figure 1).

### Postoperative Ventricular Arrhythmia

Rhythm disturbances were evaluated by continuous electrocardiographic monitoring from the time of the removal of the aortic clamp to 72 h postoperatively. POVA was defined as any premature ventricular beats, ventricular fibrillation, and bradyarrhythmias during the reperfusion period or in the postoperative period (Valderrábano et al., 2016b).

### Electrocardiographic Measurements

After a quality check assessing recording speed (>25 mm/s), standard 12-lead ECGs (MedEx-ECG workstation, Madicks Co., Ltd., BeiJing, China) were recorded in the same way and were evaluated for the QRS width, QT or QTc interval, ST-segment level at the J point, Tpeak–Tend interval (Tpe), iCEB, S_V1_+R_V5_ >35 mm, QT/QTc ratio, and fQRS. The Tpeak–Tend interval (Tpe) is defined as the interval from the peak to the end of the T-wave in the V_2_ lead; this interval was measured in three beats and then averaged (Demidova et al., 2019). The QT interval was automatically measured in lead II and corrected for the heart rate with the Bazett method. An S-wave in lead I was defined as a depth >0.1 mV and/or a width >40 ms (Calò et al., 2016). An R-wave in lead aVR was defined as an R-wave height >0.3 mV and/or an R-wave/q-wave ratio >0.75 (BabaiBigi et al., 2007). Regarding the defined short- or long-QT intervals, the current guideline recommends an upper normal limit of a corrected QT (QTc) interval of 440 ms and a lower limit of 340 ms (Schwartz and Ackerman, 2013; Campuzano et al., 2018). The presence of fQRS was defined as abnormal fragmentation within the QRS complex as four spikes in several leads as described previously. Based on the American Heart Association’s scientific statement, the J wave or J point was defined as the amplitude of the elevation that had to be at least 1 mm above the baseline level at the ST-segment level, either as QRS slurring or notching (Patton et al., 2016). Atrial fibrillation was diagnosed as previously reviewed. The voltage sum S_V1_+R_V5_ >35 mm using the Sokolow-Lyon voltage criteria was diagnosed as left ventricular hypertrophy (LVH) (Antikainen et al., 2003). iCEB was defined as QRS/QT.

### Selection of Preoperative ECG Markers

We selected 15 potential ECG markers (Figure 1) of POVA risk according to previous reviews. Four potential markers were associated with depolarization/conduction disorders, including a QRS width>120 ms, a fragmented QRS in the precordial leads (fQRS), an S wave in lead I, and an R-wave in lead aVR. Five potential markers were implicated in repolarization disorders: the J wave or J point, short- or long-QT intervals, QT/QTc, and Tpe. One potential marker was related to an imbalance between repolarization and depolarization, that is, the iCEB. The following remaining potential markers were unclassified: atrial fibrillation (AF), ST-segment change, and S_V1_+R_V5_ >35 mm representing LVH.

### Statistical Analysis

Data are expressed as the mean ± SD or median (interquartile range). Student’s *t* test was performed to compare variables between groups, and categorical data and percentage frequencies were compared with the chi-square or Fisher exact test as appropriate. Univariate analysis was performed for associated POVA events. Multivariate analysis was used to estimate the occurrence risk for those predictors and identified independent risk factors relative to POVA. Receiver operating characteristic (ROC) curve and area under the curve (AUC) analyses were used to estimate the usefulness of preoperative Tpe for the prediction of POVA. Candidate variables were defined as a *p* value < 0.10 in univariate analysis and the 2-group comparison of each model. A *p* value <0.05 was considered statistically significant.

## Results

### Population Characteristics

Altogether, 823 patients from the same hospital were included in this analysis, with women accounting for 51.3% of the entire cohort and a mean age of 54.7 years. A total of 546 patients underwent isolated valve surgery (66.3%), and 186 patients underwent isolated coronary artery bypass grafting (CABG) [22.6%]. Ventricular and auricular septal defect repair was conducted in 28 and 71 patients, respectively, and 28 patients underwent cardiac myxoma removal. In total, 44 patients underwent other isolated surgeries. POVA occurred in 338 patients (41%). Table 1 shows the characteristics of patients with and without POVA. The proportions of patients with diabetes, stroke, and undergoing CABG or valve surgery were higher among patients with POVA than among patients without POVA. Patients with ventricular and auricular septal defect were, more often, POVA free. Table 2 shows preoperative electrocardiographic data in patients with POVA and POVA-free. The proportions of patients with the presence of S-wave in lead I, R-wave in lead aVR, Tpe interval >112.5-ms, ST-segment change, J wave, Preoperative AF, and S_V1_+R_V5_ > 35 mm were higher among patients with POVA than among patients without POVA. Compared with the patients with POVA-free, the duration of QRS complex, Tpe interval, and QTc duration in the patient with POVA were prolonged.

**TABLE 1:** Baseline Characteristics in patients with POVA and POVA-free.

	POVA (*n* = 338), % (*n*)	POVA-free (*n* = 485), % (*n*)	*p* Value
Age, yrs	52.2 ± 12.8	46.8 ± 11.6	0.062
Male	48.7% (165)	33.3% (160)	<0.001
Weight, kg	58.3 ± 11	54.3 ± 11.2	0.69
NYHA class ≥ III	53.8% (182)	66% (320)	<0.001
Medical history			
Dyslipidemia	9.5% (48)	5.3% Tse et al. (2021)	0.545
Hypertension	20.5% (69)	8.9% (43)	0.91
Diabetes	5.7% Takagi et al. (2013)	5.3% Tse et al. (2021)	0.027
Stroke	12.8% (43)	2.6% Calò et al. (2016)	0.023
COPD	3.2% Valderrábano et al. (2016b)	6.2% Shenasa et al. (2015)	0.057
Previous MI	10.2% Valderrábano et al. (2016a)	4.4% Rosenthal et al. (2015)	0.28
Cardiac cardioplegia			
HTK solution	64.7% (219)	19.1% (93)	0.038
Cold blood cardioplegia	35.2% (119)	80.8% (392)	0.038
Surgery variables			
GABG	27.4% (93)	10.7% (93)	0.005
Heart valve surgery	62.1% (210)	69.1% (336)	<0.001
Ventricular septal defect repair	2.5% Erikssen et al. (2012)	4% Takagi et al. (2013)	0.005
Auricular septal defect repair	7.6% Tse et al. (2021)	9.3% (45)	<0.001
Cardiac myxoma removal	3.8% Calò et al. (2016)	3.1% (Schwartz and Ackerman, 2013)	0.18
Others	6.8% Valderrábano et al. (2016a)	0.8% Erikssen et al. (2012)	0.14

MI, myocardial infarction; COPD, chronic obstructive pulmonary disease; GABG, coronary artery bypass grafting.

**TABLE 2:** Preoperative electrocardiographic data in patients with POVA and POVA-free.

	POVA (n = 338), % (n)	POVA-free (n = 485), % (n)	*p* Value
Depolarization/conduction disorders markers			
Duration of QRS complex, ms	96.2 ± 17.7	89.3 ± 14.9	<0.001
Fragmented QRS	17.9% (61)	12.0% (58)	0.054
S-wave in lead I	29.4% (100)	26.3% (128)	<0.001
R-wave in lead aVR	95.5% (323)	86.6% (420)	<0.001
Repolarization disorders markers			
Tpe interval in lead V_2_, ms	115.1 ± 36.8	87.0 ± 24.1	<0.001
Tpe interval >112.5-ms	53.8% (182)	13.8% (67)	<0.001
QT duration, ms	382.6 ± 61.7	369.5 ± 50.1	0.024
QTc duration, ms	435.2 ± 50.1	415.6 ± 40.8	<0.001
QT/QTc ratio	0.9 ± 0.1	0.9 ± 0.1	0.486
Long- or short- QT intervals	55.7% (188)	39.7% (193)	0.189
ST-segment change	17.3% (58)	12.0% (58)	<0.001
J wave	69.2% (234)	18.7% (91)	<0.001
Abnormal depolarization-repolarization			
Combination of the J wave and fQRS	16.6% (56)	7.5% Moss et al. (2002)	<0.001
Unclassified markers			
Preoperative AF	19.8% (67)	25.4% (123)	<0.001
Preoperative VA	29.4% (100)	16.5% (80)	0.07
iCEB (QRS/QT)	0.3 ± 0.1	0.2 ± 0.1	0.081
Pathological Q wave	4.4% Schwartz and Ackerman, (2013)	0.8% (Morita et al. (2008)	0.092
S_V1_+R_SV5_ > 35 mm	16.6% (56)	4.9% Panikkath et al. (2011)	<0.001
Heart rate, bpm	80.8 ± 19.9	79.5 ± 19.3	0.485

AF, atrial fibrillation; VA, ventricular arrhythmia; iCEB, the index of cardiac electrophysiological balance.

### Univariate Analysis in Preoperative ECG

Table 3 shows the results of the univariable analysis. In univariate analysis, heart rate, fQRS, the S wave in lead I, QT/QTc, ST-segment change, and preoperative AF did not predict POVA. Preoperative VA, the R-wave in lead aVR, QRS duration, iCEB, QT interval, QTc interval, Long- or short- QT intervals, Tpe in lead V_2_, inferolateral J wave, J wave + fQRS, Q wave, and S_V1_+R_V5_ > 35 mm were associated with POVA.

**TABLE 3:** Analyses of independent electrocardiographic markers of POVA using logistic regression models.

Variables	Univariate		Multivariable	
	Adjusted OR (95% CI)<	*p* Value	Adjusted OR (95% CI)	*p* Value
J wave	9.75 (6.04–15.72)	<0.001	3.80 (1.88–7.66)	<0.001
Tpe >112.5 ms	7.26 (4.43–11.89)	<0.001	2.80 (1.57–4.99)	<0.001
S_V1_+R_SV5_ > 35 mm	3.87 (1.85–8.10)	<0.001	2.92 (1.29–6.60)	0.01
Preoperative VA	2.11 (1.29–3.45)	0.003	—	—
R-wave in lead aVR	3.29 (1.40–7.70)	0.006	—	—
QRS duration	0.97 (0.96–0.98)	<0.001	—	—
ICEB	0.057 (0.002–1.51)	0.087	—	—
QT interval	0.99 (0.992–1.00)	0.027	—	—
QTc interval	0.99 (0.985–0.995)	<0.001	—	—
Combination of the J wave and fQRS	2.43 (1.27–4.66)	0.007	—	—
Q wave	5.21 (1.06–25.4)	0.041	—	—
Heart rate, bpm	0.99 (0.98–1.00)	0.484	—	—
FQRS	1.59 (0.89–2.83)	0.11	—	—
the S wave in lead I	1.16 (0.74–1.84)	0.500	—	—
QT/QTc	1.94 (0.29–12.65)	0.486	—	—
ST-segment change	1.52 (0.85–2.72)	0.151	—	—
Preoperative AF	0.72 (0.44–1.19)	0.206	—	—

### Multivariate Analysis of Preoperative ECG Parameters and Receiver Operating Characteristic Analysis

The multivariate logistic regression analysis identified the following preoperative factors that could predict POVA: the J wave (AOR: 3.80; 95% CI: 1.88–7.66; *p* < 0.001), and S_V1_+R_V5_ > 35 mm (AOR: 2.92; 95% CI: 1.29–6.60; *p* = 0.01). Each 10-ms decrease in Tpe was associated with a 16% increase in the risk for POVA (AOR: 0.984; 95% CI: 0.97–0.99; *p* < 0.001). In addition, ROC curve analysis revealed the utility of Tpe for the prediction of POVA. The AUC was 0.724 (95% CI 0.67–0.77, *p* < 0.0001), with discrimination at 112.5-ms yielding a sensitivity of 54.5% and specificity of 87.5% (Supplementary Figure S1). Subsequently, multivariate analysis again identified that preoperative Tpe >112.5 ms was independently associated with POVA (AOR: 2.80; 95% CI: 1.57–4.99; *p* < 0.001).

### Construction and Validation of a POVA Risk Prediction Model

**FIGURE 2:**
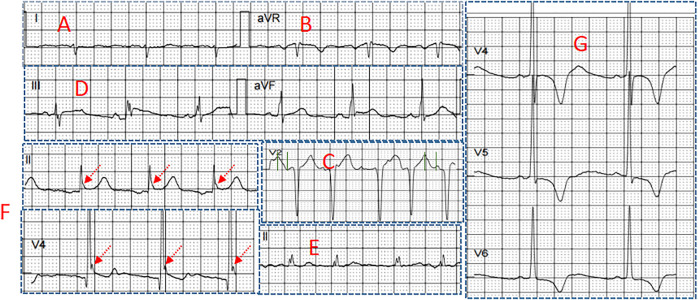
Several electrocardiographic markers for POVA **(A)** S-wave in lead I **(B)** R-wave in lead aVR **(C)** Tpeak-Tend interval in lead V_2_ **(D)** Combination of fragmented QRS and J wave **(E)** fragmented QRS **(F)** J wave in the inferior leads, and (G) S_V1_+R_V5_ >35 mm.

Points were assigned to each risk predictor according to its number, enabling the development of a model that predicted the risk of POVA. The presence of the J wave was assigned 1 point, the presence of the S_V1_+R_V5_ > 35 mm wave was assigned 1 point, and the presence of the Tpe >112.5-ms wave was assigned one point. Thus, the POVA risk prediction model was developed with a range of 0–3 points based on this calculation. The rates of POVA and the risk scores in the cohorts are shown in Figure 2. In addition, the established ROC curve and AUC were validated in the model for the total cohort (Supplement Figure 1). The AUC for this model was 0.797 (*p* < 0.0001).

## Discussion

The present study showed the following findings (El-Chami et al., 2012): a preoperative J wave, S_V1_+R_V5_ >35 mm, and Tpe >112.5 ms were independently associated with POVA, and (Valderrábano et al., 2016a) the risk prediction model could preoperatively predict the risk of POVA.

### Preoperative Electrocardiographic Markers of POVA.

A preoperative J wave was independently associated with POVA. There is a long-established concept that the J wave is benign. This concept has been significantly contradicted in recent years. Some studies have shown that the presence of the J wave was associated with a higher incidence of ventricular tachyarrhythmia (VT/VF) in patients with Brugada syndrome (Takagi et al., 2013), short QT syndrome, noncompaction cardiomyopathy (Caliskan et al., 2012), and ischemic heart disease (Patel et al., 2012). This evidence implies that more arrhythmogenic susceptibility appears in patients with the presence of the J wave. Under surgical conditions and global cardiac ischemic factors, there is a general risk of POVA in patients. Our finding that a preoperative J wave was more common in patients experiencing POVA is in agreement with these previous findings. The underlying mechanism may be that the disparity of outward Ito current density between segments of the ventricular epicardium has the potential to cause ventricular tachyarrhythmias.

In addition, Tpe, representing cardiac repolarization inhomogeneity, was implicated in POVA, and ROC curve and multivariate analyses considered that Tpe >112.5 ms was independently associated with POVA. Todd et al. suggested that the T-peak to T-end interval predicts ventricular tachyarrhythmia in a primary prevention population with systolic cardiomyopathy (Rosenthal et al., 2015). The T peak-Tend interval was previously associated with endothelial dysfunction, arterial stiffness and impaired coronary perfusion (Mozos, 2015). Ragesh et al. considered that a prolonged Tpeak-to-Tend interval on resting ECG was associated with increased VF for sudden cardiac death (Panikkath et al., 2011). Tpeak-Tend interval was reported to be prolonged in hypertensive, overweight and obese patients, smokers, and persons with prolonged exposure to shift work (Mozos and Filimon, 2013). Some studies suggested that repolarization variability predict ventricular arrhythmogenesis in mouse or human hearts (Castro-Torres et al., 2015; Tse et al., 2021). Our finding is in accordance with previous reviews of Tpe predicting cardiac arrhythmias. The ventricular myocardium includes three electrophysiologically distinct cell types-endocardial, epicardial, and subendocardial M cells. Tpe corresponds to the transmural dispersion of repolarization in the ventricular myocardium, a period during which the epicardium has repolarized and is fully excitable, but the M cells are still in the process of repolarization and are vulnerable to the occurrence of early afterdepolarizations. If conditions permit, these early afterdepolarizations can lead to reentry, resulting in polymorphic ventricular tachycardia or ventricular fibrillation (Antzelevitch, 2007).

S_V1_+R_V5_ >35 mm was considered a risk marker of POVA. S_V1_+R_SV5_ >35 mm represents left ventricular hypertrophy (LVH) according to the Sokolow-Lyon ECG criteria. Seth R. et al. found that ECG LVH was an independent predictor of nonoperative arrhythmic events (Bender et al., 2012). A previous review suggested that left ventricular hypertrophy (LVH) poses an independent risk of increased morbidity and mortality, including atrial arrhythmias, ventricular arrhythmias, and sudden cardiac death (Shenasa et al., 2015). The gap junction surface area is reduced, and its unusual distribution may decrease the threshold of arrhythmogenesis in patients when undergoing cardiac surgery (Wolk, 2000).

### A POVA Risk Prediction Model

**FIGURE 3:**
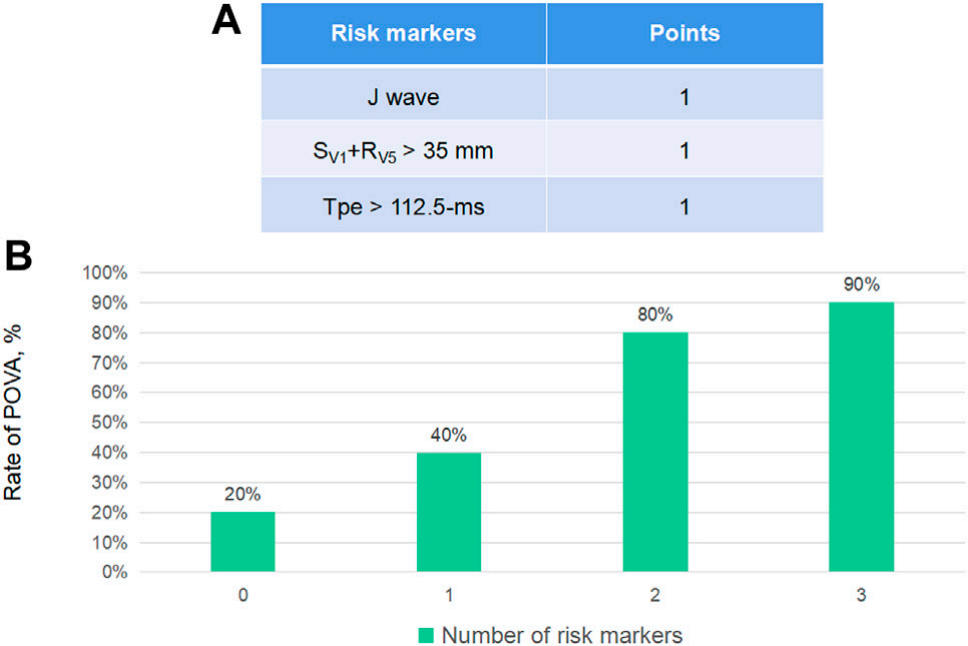
Electrocardiographic markers for POVA and the development of the POVA risk score model, According to the number of risk markers, we assigned points **(A)** and subsequently developed a model predicting POVA **(B)**.

According to the number of risk markers, we assigned points and subsequently developed a model predicting POVA. The POVA risk prediction model was developed with a range of 0–3 points based on this calculation in Figure 3A. The rates of POVA and its risk scores in the cohorts were the highest in the two-point position and did rise by points increased in Figure 3B. Subsequently, ROC curve analysis was performed for the validation of the model. The AUC for this model was 0.797 (*p* < 0.0001). The model is simple, efficient and practical. Patients with ventricular and auricular septal defect were, more often, POVA free. There is rarely the relationship between some types of operation including ventricular and auricular septal defect and postoperative arrhythmia. Potential reasons probably were that the patients undergoing the mentioned types of surgery were younger.

### Prediction Factors and Clinic Outcomes of a POVA

An abundant study suggested that POVA is associated with increased short- and long-term mortality after cardiac surgery (CS). Previous studies have suggested that have shown that POVA predicts higher (21.7–28.9%) in-hospital mortality compared with control (1.4–1.9%) (Ascione et al., 2004; Yeung-Lai-Wah et al., 2004). Besides, Most deaths in patients with POVA occur in the hospital and within the first year after discharge (El-Chami et al., 2012). Rodrigo et al. have suggested that developing a new POVA increases the risk of in-hospital mortality independently in the medical intensive care unit (Valderrábano et al., 2016a). Older age, female sex, systemic hypertension, peripheral vascular disease (PVD), lower ejection fraction (EF), and emergent surgery are associated with a higher risk of POVA, whereas off-pump surgery seems to be protective (Yeung-Lai-Wah et al., 2004; El-Chami et al., 2012). Some studies looking at risk of POVA after CS found that patients with POVA are older than patients without POVA (Steinberg et al., 1999). Ascione et al. reported a trend toward a reduction in POVA with the use of off-pump surgery compared with on-pump. A group of patients with LV dysfunction associated with a previous MI have been reported that common POVA (Moss et al., 2002). The presence of PVD in some studies is found to be predictive of POVA and operative mortality (Loponen et al., 2002; Bonacchi et al., 2020). Our previous study has been suggested that systemic hypertension was an independent risk factor of post-operative ventricular fibrillation. The need for emergent CABG also strongly predicted POVA. Emergency CABG generally carries a higher risk of in-hospital mortality and added requirements for hemodynamic support (Yang et al., 2005).

### Limitations

These electrocardiographic markers are dynamic; thus, the true prevalence of this coexistence is difficult to evaluate. Various methods to measure the Tpe interval have been used in other studies. Our results may not be reproducible with other Tpe measurement methods. In addition, limits of the Bazett formula and the heterogeneity of the studied population also were considered necessarily. This retrospective study evaluated patients from a single hospital, existing the observational bias. There is the limited clinical applicability of the findings in the absence of other clinical and biochemical markers.

## Conclusion

The ECG biomarkers including J wave, Tpe >112.5 ms, and S_V1_+R_SV5_ >35 mm were significantly predicted POVAs. A risk predicting model developed with electrocardiographic risk markers preoperatively predicted POVAs.

## Data Availability

The raw data supporting the conclusion of this article will be made available by the authors, without undue reservation.
