# Supplementary figures and images for: Electrocardiography is Useful to Predict Postoperative Ventricular Arrhythmia in Patients Undergoing Cardiac Surgery: A Retrospective Study
Source: Front Physiol. 2022 May 2;13:873821. doi: 10.3389/fphys.2022.873821 (PMC9108335; doi:10.3389/fphys.2022.873821)

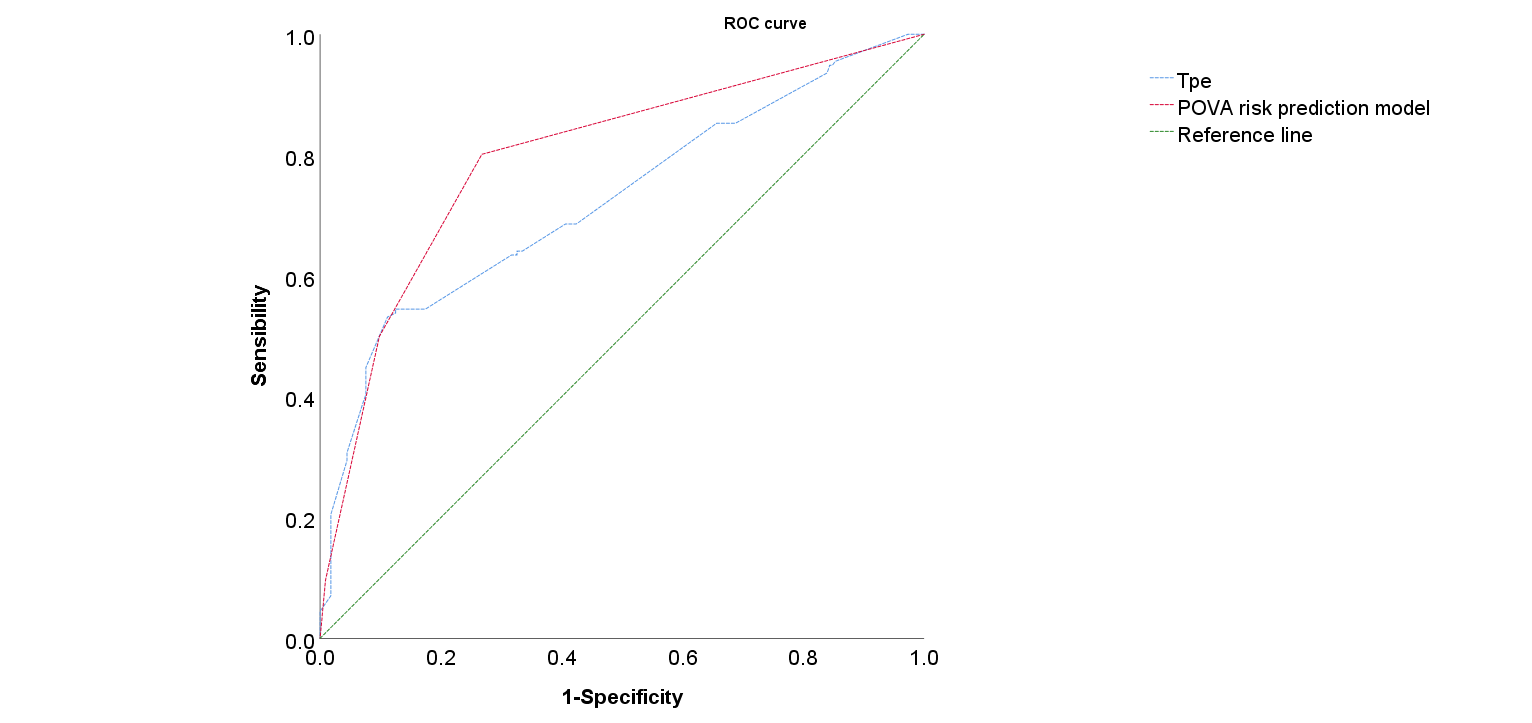

Supplement: Supplementary file 1 [file Image1.tif]
